# Quinine in Malaria

**Published:** 1894-06-02

**Authors:** Herbert W. G. Macleod

**Affiliations:** late H.M. Bengal Army


### EDITOR'S LETTER-BOX.

[Our correspondents are reminded that proxility is a great bar to publication, and that brevity of style and conciseness of statement greatly facilitate early insertion.]

### QUININE IN MALARIA.

Sir,—I notice that the writer of the excellent article on Malaria, appearing in your paper (*vide* The Hospital, pp. 27 and 94), mentions that quinine acts as a specific in this cachexia. Having myself treated several scores of such cases, I may venture to say that although quinine and the cinchona series are without doubt preventives against malaria, and are also of service in early attacks, they are practically of no use at a later stage of the disease. Arsenic, on the contrary, is of the greater service all through. I can vouch for the fact that exhibited as Fowler's solution, or as the hydrochlorate, this agent not only cuts short malarial fever when early treated, but also reduces the size of "ague-cake." Having myself been a frequent sufferer from malarial neuralgia, I am able to say that quinine has practically failed whereas arsenic has succeeded. It need not be given only as an anti-malarial remedy, but also as a "nervine tonic," whatever that may mean. I have used it in various combinations—generally with iron and a bitter—and have, as a rule, had excellent results. One case in particular of intense sciatica, occurring in a cavalry officer, was greatly alleviated by taking Fowler's solution in large doses at stated times, and with certain precautions. With this internal treatment, assisted with local applications, the sciatica was entirely cured. One thing, however, must be avoided, and that is the exhibition of very small doses. They will have little effect. On the contrary, the patient must be "educated up to the limit of his toleration." This, of course, varies in each case.

Only quite recently a gentleman in London suffering from severe neuralgia (caused apparently by over-strain and much mental work) informed me that a few doses of arsenic with a local anæsthetic, which I ordered, immediately cut short the attack. His was certainly not a case of malarial poisoning. In conclusion, I may add that so high an authority as Surgeon-General Sir William Moore (late of the Bengal Army) does not think quinine is of much use in malaria.—I am, dear sir, yours truly,

Herbert W. G. Macleod, M.D., M.C.Edin.

(late H.M. Bengal Army).

